# 1,25-Dihydroxyvitamin D Induces a NURR1–Tyrosine Hydroxylase Transcriptional Axis Modulated by Rexinoid/RXR Signaling in Parkinson’s Disease-Relevant Human Neural Cell Models

**DOI:** 10.3390/cells15131210

**Published:** 2026-07-03

**Authors:** Michael A. Sausedo, Sanchita Mallick, Zhela L. Sabir, Sarah Livingston, Quang T. Nguyen, Mobin Emran Doost, Carl E. Wagner, Pamela A. Marshall, Carol A. Haussler, Mark R. Haussler, Peter W. Jurutka

**Affiliations:** 1 School of Mathematical and Natural Sciences, Arizona State University, Glendale, AZ 85306, USA; msausedo@asu.edu (M.A.S.); sanchita.mallick@gmail.com (S.M.); zhelasa@gmail.com (Z.L.S.); sarahklivingston5@gmail.com (S.L.); qtnguye4@asu.edu (Q.T.N.); carl.wagner@asu.edu (C.E.W.); pamela.marshall@asu.edu (P.A.M.); 2 College of Medicine, University of Arizona, Phoenix, AZ 85004, USA; medoost@arizona.edu (M.E.D.); chaussle@arizona.edu (C.A.H.); haussler@arizona.edu (M.R.H.); 3 John Shufeldt School of Medicine and Medical Engineering, Arizona State University, Phoenix, AZ 85004, USA

**Keywords:** vitamin D, tyrosine hydroxylase, NURR1, Parkinson’s disease, rexinoids, RXR/VDR, dopaminergic neurons, gene expression

## Abstract

The hormonal vitamin D metabolite, 1,25-dihydroxyvitamin D (1,25D), produced primarily in the kidney, acts in numerous end-organs via the nuclear vitamin D receptor (VDR) to trigger molecular events that orchestrate bone mineral homeostasis, immune responsiveness, and aspects of behavior. Tyrosine hydroxylase (TH) encodes a neuronally expressed enzyme that catalyzes the initial, rate-limiting step in the production of several catecholamine neurotransmitters and hormones, including dopamine, norepinephrine, and epinephrine. Herein we report that *TH* mRNA is significantly induced (2.5-fold) and *NURR1* mRNA is induced 9.3-fold by 10 nM 1,25D in differentiated human SH-SY5Y neuroblastoma cells. Similar results were observed in human U87 glioblastoma cells (*TH*, 2.6-fold; *NURR1*, 3.6-fold). Comparative analysis of *TH* gene promoter-proximal sequences from human, mouse, and rat identifies candidate NURR1-responsive elements (NBREs) at the following positions: −35, −855, −1470, and −2343 bp in the human gene; −34 and −961 bp in the mouse gene; and −34, −350, and −873 bp in the rat gene, consistent with NURR1 acting as a recurring regulatory factor at *TH* promoters across mammalian species. Furthermore, by interrogating VDR ChIP-seq/cistrome datasets, we identified candidate vitamin D-responsive elements (VDREs) at the human NURR1 locus that provide a plausible genomic framework for direct regulation of NURR1 by 1,25D/VDR. We propose that 1,25D-liganded VDR acts as a primary inducer of *NURR1*, which in turn secondarily activates expression of the *TH* gene, thereby defining a transcriptional route through which 1,25D/VDR signaling may influence TH-linked dopaminergic gene programs. Retinoid X receptor (RXR) may facilitate both NURR1-dependent and -independent potentiation of *TH* transcription because the rexinoid, bexarotene, significantly enhances *TH* mRNA in human U87 cells, either alone (2.0-fold) or in combination with 1,25D (4.1-fold). In addition, bexarotene and its novel analogs, **A41** and **A55**, induce *NURR1* mRNA expression in U87 cells by 2.8-, 3.1-, and 4.8-fold, respectively, with **A55** outperforming the parent compound at matched concentration. Because Parkinson’s disease is characterized by the selective degeneration of dopaminergic neurons and impaired NURR1-dependent transcriptional programs, our findings identify a 1,25D/VDR–NURR1–RXR transcriptional axis as a previously underappreciated regulatory framework for studying *TH* gene expression and dopaminergic gene regulation in Parkinson’s disease-relevant neural contexts.

## 1. Introduction

The active hormonal vitamin D metabolite, 1,25-dihydroxyvitamin D (1,25D), acts through the vitamin D receptor (VDR) and is increasingly recognized as a pleiotropic neuroactive hormone with roles in neuronal differentiation, neurotransmitter regulation, cognitive function, oxidative stress, and neuroinflammatory signaling [1,2,3,4,5,6]. VDR is expressed in multiple regions and cellular compartments of the brain, including neuronal stem cells, mature neurons, astrocytes, and oligodendrocytes, supporting the possibility that 1,25D/VDR signaling contributes to both neurodevelopmental and mature CNS functions [2,3,4,5]. Immunohistochemical detection of VDR in the substantia nigra region of the CNS suggests that 1,25D plays a part in the actions of dopamine neurons and animal models for Parkinson’s disease (PD) reveal that vitamin D attenuates the death of dopaminergic neurons, leading to an enhancement of dopamine production [3]. In summary, potential novel effects of vitamin D in the CNS are of great current interest and have been comprehensively reviewed. Recent updates by Cui and Eyles [6] further highlight vitamin D as a regulator of neurodevelopment, neurotransmitter synthesis, oxidative stress, and neuroinflammation, reinforcing its relevance to neurological disease. Similarly, Lasoń and colleagues have specifically positioned the VDR as a candidate therapeutic target in age-related neurodegenerative disorders, particularly Parkinson’s and Alzheimer’s diseases, integrating the receptor’s neuroprotective, anti-inflammatory, and pro-survival actions into a translational rationale for vitamin D-based interventions [7].

Herein, we examined regulation of tyrosine hydroxylase (TH) by 1,25D/VDR in human neural cell lines. TH is the rate-limiting enzyme in the synthesis of catecholamine neurotransmitters/hormones from tyrosine, namely dopamine in dopaminergic neurons, norepinephrine in norepinephrinergic neurons, and epinephrine in adrenal medulla [8]. Studies by Zhou et al. further emphasize that dysregulation of the TH–dopamine axis is central to PD pathogenesis, underscoring the importance of understanding mechanisms that control *TH* gene expression [9]. *TH* does not possess proven vitamin D-responsive elements (VDREs), yet the expression of the *TH* gene is induced by 1,25D [10]. The molecular mechanism through which 1,25D/VDR activates the expression of the *TH* gene is not understood at present, but the potential for the vitamin D hormone to enhance dopamine in the CNS is of great interest.

Consistent with this biological rationale, clinical and translational studies have linked inadequate vitamin D status with PD risk and progression, although supplementation outcomes have been variable [11,12]. Genetic studies have also implicated VDR variation, including the FokI (rs2228570) polymorphism, as a potential contributor to PD susceptibility [12,13].

These clinical and genetic associations motivate a closer look at the dopaminergic neurons themselves and the molecular machinery that establishes their identity. Dopamine is responsible for motor functions and emotions, with a primary source of dopamine being mesodiencephalic dopaminergic neurons (mdDAs), located in the substantia nigra pars compacta and ventral tegmental area (VTA). Collectively, mdDAs of the VTA and of the retrorubral field participate in the regulation of emotions and reward. During early neurogenesis triggered by neurogenin, mdDA predecessors begin to express NURR1, an orphan nuclear hormone receptor that is required for the genesis of mdDA neurons and that is a signature for the postmitotic phase of mdDA development. Next, reflecting the maturation of mdDA neurons, paired-like homeodomain transcription factor 3 (Pitx3) emerges. NURR1 regulates proteins governing dopamine synthesis and transport, whereas reliance on Pitx3 occurs in a subpopulation of mdDA neurons that eventually produces the mature substantia nigra pars compacta. Importantly, studies by Kim et al. demonstrate that pharmacologic activation of NURR1 produces disease-modifying effects in PD models, highlighting its therapeutic potential [14]. Beyond its role in dopaminergic neuron preservation, NURR1 also functions as an endogenous suppressor of pro-inflammatory gene expression in microglia and astrocytes, and its dysregulation in PD contributes to the chronic neuroinflammation that accompanies dopaminergic degeneration, broadening the rationale for NURR1-targeted therapeutic strategies [15]. In vivo, NURR1 and Pitx3 have been reported to function in concert at the genomic level to control the transcription of genes involved in dopamine metabolism [16]. A combination of genomic and epigenetic regulation orchestrates induction of the *TH* gene. Epigenetic complexes acting at upstream CpG islands and local co-repressor complexes associating with NRSF- and KAISO-binding motifs in the *TH* promoter region suppress the classic transcription factor-activating complexes consisting of CREB and NURR1 [17]. These trans-activators awaken when one specific CpG (−1868 bp from the *TH* transcription start site) is demethylated, leading to the dissociation of NRSF- and KAISO-bound co-repressors and accompanying histone acetylation. As a result, CREB and NURR1 induce *TH* gene expression, and induction of NURR1 itself could further boost *TH* mRNA via a mechanism analogous to that of prolonged depolarization, which elicits enhanced NURR1 binding to NGFI-B response elements (NBREs) and modifies histone methylation and acetylation patterns [17].

*TH* gene expression is also influenced by broader developmental and chromatin-regulatory mechanisms involving FGF2, nuclear FGFR1 (nFGFR1), CREB-binding protein (CBP), and additional transcriptional regulators that coordinate cell-specific TH control in the brain [18]. These regulatory concepts are relevant here because NURR1, CREB, RXR-linked signaling, and chromatin-modifying co-regulators may converge at TH regulatory regions to modulate transcriptional output [18].

Acutely, major regulation of TH activity is exerted by phosphorylation cascades and alternative posttranslational modifications. With respect to chronic control of *TH* mRNA, glucocorticoids (stress), drugs (e.g., cocaine), and second messengers such as cAMP enhance *TH* gene transcription. Pertinent to the present study, *TH* mRNA has been shown to be induced by 1,25D in both normal bovine adrenal medulla [19] and human SH-SY5Y neuroblastoma [10] cells (a model for dopaminergic neuronal cells). Herein, we have sought to extend these findings of 1,25D as a possible potentiator of dopamine synthesis in the CNS, analogous to our previous findings that 1,25D induces tryptophan hydroxylase-2 (TPH2) [20] but represses serotonin reuptake transporter and monoamine oxidase-A [21] in serotonergic neuronal cells, thereby amplifying serotonin concentrations. A goal of the present research was to accumulate additional data on the induction of TH by 1,25D in order to identify trans-factor candidates that may mediate this action of the vitamin D hormone. Notably, Eyles and colleagues have already put forth N-cadherin as a candidate secondary mediator of TH induction by 1,25D [10]. In the current report, we propose that NURR1 is an equally suitable candidate to serve as the secondary mediator of TH induction by 1,25D/VDR.

## 2. Materials and Methods

### 2.1. Mammalian Cell Culture

Two human cell lines (from the American Type Culture Collection, Manassas, VA, USA) were employed in this study. Human brain glioblastoma/astrocytoma (U87 MG) cells were grown in DMEM/high glucose, L-glutamine, and pyruvate (Hyclone Laboratories, Logan, UT, USA). Human neuroblastoma (SH-SY5Y) cells were grown in DMEM-F12 supplemented with 10% FBS. In some experiments, SH-SY5Y cells were differentiated toward a dopaminergic neuronal phenotype using a standard all-trans retinoic acid (RA) protocol. Briefly, cells were plated at sub-confluent density (~30–40%) and switched to differentiation medium consisting of DMEM-F12 supplemented with 1% FBS and 10 µM all-trans retinoic acid (Sigma-Aldrich, St. Louis, MO, USA). Cells were maintained in differentiation medium for 5–7 days with media replenished every 48 h, during which they acquired the morphological hallmarks of differentiation, including reduced proliferative activity and extension of neurites. Following differentiation, cells were dosed with 1,25D or vehicle (ethanol) for 24 h prior to RNA harvest, as described in Section 2.2. The cells were passaged/plated in the indicated media and grown in a humidified atmosphere at 37 °C and 5% carbon dioxide. The active hormonal form of vitamin D (1,25-dihydroxyvitamin D) was obtained from Enzo Life Sciences (Farmingdale, New York, NY, USA). RXR agonists (rexinoids), including bexarotene and analogs **A41**/**A55,** were synthesized in the laboratory of Dr. Carl Wagner, according to published synthetic procedures [22,23]. **A41** and **A55** have lower EC50 values and similar RAR crossover activity compared to bexarotene, as well as similar low cytotoxicity profiles.

### 2.2. Total RNA Isolation, cDNA Synthesis, and mRNA Expression Analysis via Quantitative Real-Time PCR

Cells were plated at 500,000–900,000 cells per well in a 6-well plate and treated for 24 h with either an ethanol (EtOH) vehicle control or 10 nM 1,25D. Total RNA was isolated from each well using an Aurum Total RNA Mini kit (Bio-Rad, Hercules, CA, USA) according to the manufacturer’s instructions. The RNA quantity and quality were assessed using A260/280 spectrophotometry. DNase-treated RNA (1 µg) was reverse transcribed using an iScript cDNA Synthesis kit (Bio-Rad) to produce 20 µL of first strand cDNA. For real-time PCR, 2 µL of cDNA was used in a 10 µL PCR reaction containing 5 µL FastStart Universal SYBR Green Master Mix + Rox (Roche Applied Science, Indianapolis, IN, USA) and primers. Reactions were performed in 96-well PCR plates in a Bio-Rad CFX96 instrument using a standard 40-cycle profile. Data were analyzed using the comparative ΔΔCt method as a means of relative quantitation, normalized to an endogenous reference (GAPDH) and relative to a calibrator (normalized Ct value from vehicle-treated cells) and expressed as 2^−ΔΔCt^ according to Applied Biosystems’ User Bulletin 2: Rev B, “Relative Quantitation of Gene Expression.” Primer sets for the PCR were as follows:

Human GAPDH (forward 5′-ACAACTTTGGTATCGTGGAAGGAC-3′,
reverse 5′-CAGGGATGATGTTCTGGAGAGC-3′);
Human TH (forward 5′-GTGCCGGGCTGCTGTCCTCCTACGGG-3′,
reverse 5′-CACGGGCTGTCCAGCACGTCGATCCC-3′);
Human NURR1 (forward 5′-CGACATTTCTGCCTTCTCC-3′,
reverse 5′-GGTAAAGTGTCCAGGAAAAG-3′).

For real-time PCR results, data are expressed as means ± SD. All data are presented as fold-effects, with transcription of the gene in question (i.e., mRNA level) in the absence of 1,25D (i.e., ethanol vehicle alone) set at 1.0-fold. Thus, all results are normalized to basal transcription and presented graphically and numerically as fold-effects of the single tested variable, i.e., 1,25D. Because the design of the experiments was a simple motif in which the ability of 1,25D to enhance transcription over basal levels (i.e., ethanol vehicle) was assessed, statistical differences between two groups were determined by a two-tailed Student’s *t*-test. However, because variance was high in several experiments, further statistical analyses were performed using GraphPad Prism (v. 9.0) to generate ANOVA data, followed by a post hoc Dunnett test that compares every mean to a control mean and considers the scatter of all the groups. In each figure illustrating fold-effects of 1,25D, exact *p*-values (unless <0.0001) are listed above the bars for comparisons between concentrations of 1,25D and EtOH control, except when the difference from the vehicle control is not significant (NS); a *p*-value of less than 0.05 is considered significant. Unless otherwise indicated in a specific figure legend, qPCR experiments were performed using at least 3–5 independent biological replicates, with 3–4 technical replicate reactions per treatment condition.

### 2.3. NBRE Luciferase Assay

At time zero, U87 cells were plated into 24-well cell culture plates at a cell density of 40,000 cells/well 18–24 h before transient transfection (Fisher Scientific, Waltham, MA, USA). The appropriate plasmid DNAs were introduced to the cells using a polyethyleneimine (PEI) transfection reagent (Fisher Scientific). The cells in each well received 2 μL/well of PEI reagent, 20 ng/well of pRL-null (the Renilla control plasmid), and 250 ng of a firefly luciferase plasmid which included the NBRE sequence. In addition, 50 ng of pSG5-hVDR and/or pSG5-hNURR1 expression vector were utilized in a subset of experiments. Plasmids were obtained commercially or constructed in house and purified, followed by confirmation via DNA sequencing.

After 22–24 h of transfection, the cells were dosed with the negative (vehicle) ethanol (EtOH) control, 1,25D, and/or the appropriate RXR agonist. All the compounds were solubilized in ethanol to acquire the targeted concentration of vitamin D and/or rexinoid. The concentrations ranged between 1 nM and 100 nM, as indicated in the various data figures.

After 18 to 24 h of treatment with the appropriate ligands as indicated, the cells were lysed in 1X passive lysis buffer (Promega Corporation, Madison, WI, USA). The whole-cell lysates were analyzed for both firefly and *Renilla* luciferase using a Dual-Luciferase Reporter Assay Kit (Promega Corp., Madison, WI, USA) according to the manufacturer’s protocols (Promega) in a Sirius FB12 luminometer (Berthold Detection Systems, Pforzheim, Germany). In order to account for cytotoxicity, cell death, and transfection efficiency from the ligand treatment, the data obtained were normalized by dividing the firefly luciferase luminescence by the *Renilla* luciferase luminescence. The activity of the reporter gene was measured in comparison to the negative control EtOH set to 100%. Unless otherwise indicated in a specific figure legend, luciferase reporter experiments were performed using at least three independent biological replicates, with 3–4 technical replicate wells per treatment/transfection condition. Normalized luciferase data are presented as mean ± SD. Statistical comparisons were performed using two-tailed Student’s *t*-tests for two-group comparisons or one-way ANOVA followed by Dunnett’s post hoc test for multiple comparisons to the appropriate control group, with exact *p*-values reported where possible and *p* < 0.05 considered statistically significant.

## 3. Results and Discussion

**Figure 1:**
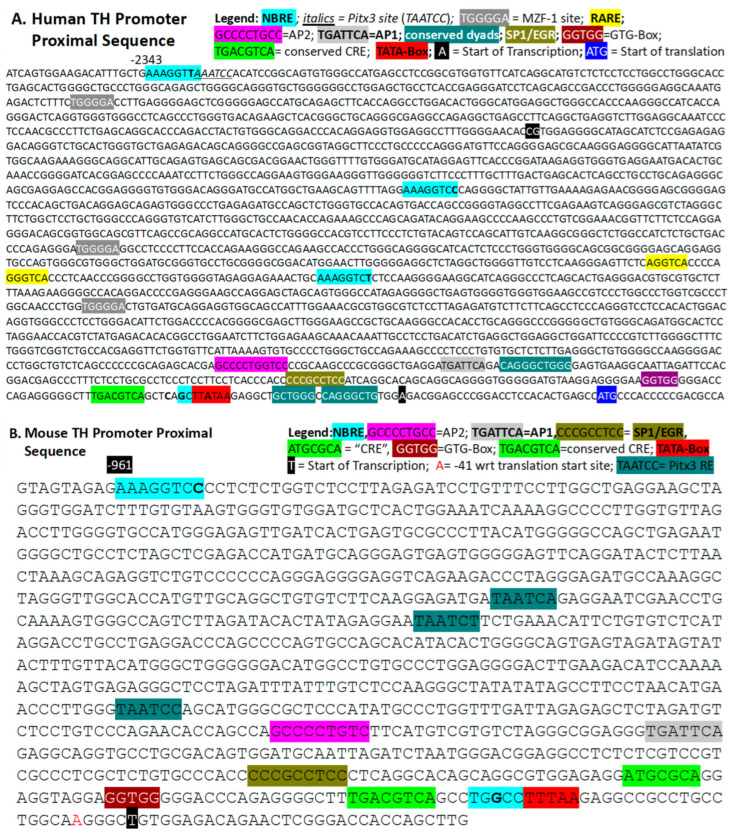

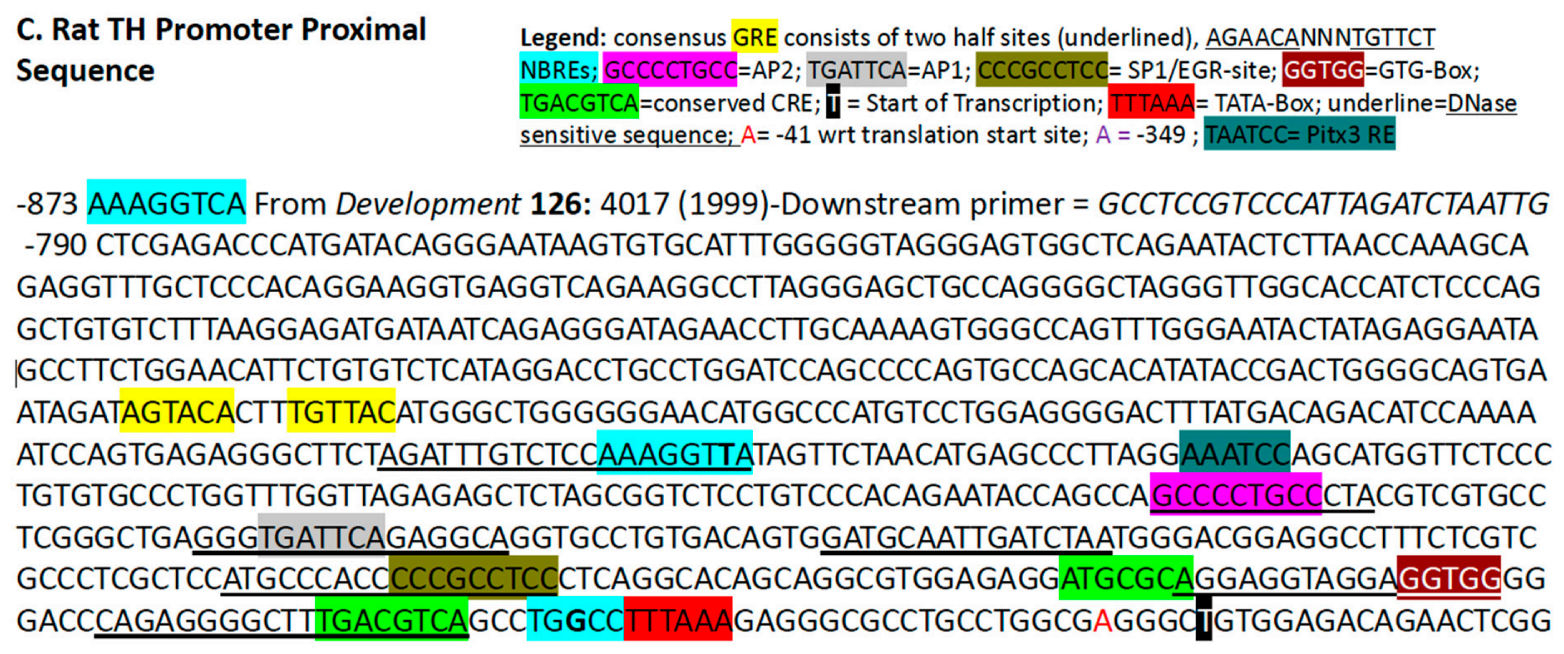
Comparison of promoter-proximal DNA sequences of the *TH* gene. Promoter-proximal DNA sequences of the *TH* gene are shown for (**A**) human, (**B**) mouse, and (**C**) rat, with putative and previously characterized transcription factor response elements annotated as indicated in the embedded key accompanying each panel. The figure highlights multiple NURR1/NBREs within the *TH* promoter regions, including sites at approximately −2343, −1470, −855, and −35 bp in the human *TH* gene; −961 and −34 bp in the mouse *Th* gene; and −873, −350, and −34 bp in the rat *Th* gene. The most proximal NBRE overlaps or lies immediately adjacent to the TATA box in all three species, representing the only positionally conserved NBRE across human, mouse, and rat, whereas the more distal promoter-proximal NBREs are not rigorously conserved among species. Additional annotated regulatory motifs include Pitx3 response elements, AP-1, AP-2, SP1/EGR, GTG-box, and conserved CRE-like elements located in the promoter region upstream of the transcription start site. In the human promoter, a putative DR5-type retinoic acid response element (RARE; AGGTCAn5GGGTCA) is indicated near −911 bp, consistent with reported retinoic acid responsiveness of *TH* transcription. In the rat promoter, a consensus glucocorticoid response element (GRE) is also shown, consistent with known stress/glucocorticoid regulation of *TH* gene expression. Underlined regions denote previously described DNase-sensitive sequences that overlap conserved regulatory domains. Collectively, the comparison emphasizes that although the precise positions of most proximal promoter NBREs differ across species, all three mammalian *TH* gene promoters contain candidate or previously characterized NURR1/NBRE-like sequences positioned within regulatory regions that may contribute to *TH* gene transcriptional control. Importantly, the comparative analysis should not be interpreted as demonstrating that each annotated NBRE is functionally active in each species; rather, it identifies candidate motifs and places them in the context of prior functional studies of NURR1-responsive *TH* gene regulatory elements.

The nucleotide sequence of the proximal promoter region of the *TH* gene in the human is depicted in Figure 1A; we have been unable to locate any canonical VDREs in this sequence or in up to 11 kb of 5′ upstream nucleotides in silico. Prominent in the sequence are a TATA box at −30 bp in relation to the start of transcription and a CRE/AP-1 site at −40 bp that is presumed to dock nFGFR1 which cooperates with NURR1 in a postulated mechanism for the postmitotic development of mesencephalic dopaminergic neurons [24]. Also occurring in the proximal promoter sequence of human *TH* are potential NURR1/NBREs at −2343, −1470, −855 and −35 bp, with the latter NBRE overlapping the TATA box. Moreover, several of these NBREs lie in the vicinity of a Pitx3 element, as NURR1 (an orphan nuclear receptor) and Pitx3 (a homeodomain-containing factor) have been identified as two key candidate transcription factors that may determine and maintain the cell fate of dopaminergic neurons [25]. At −119 bp appears an SP1 site, and at −204 and −237 bp appear AP1 and AP2 sites, respectively. At −911 bp lies a potential DR5-type retinoic acid responsive element (RARE), AGGTCAn5GGGTCA, which is consistent with the known stimulation of *TH* gene expression by retinoic acid [18].

Comparative analysis of *TH* proximal promoter sequences from human (Figure 1A), and *TH* sequences from mouse (Figure 1B) and rat (Figure 1C) reveals highly homologous regions that overlap DNase-sensitive sequences including AP-2, AP-1, SP-1, and CRE sites immediately 5′ of the TATA box and within the nucleotide realm 3′ of the glucocorticoid-responsive element that mediates the stress response of *TH* gene expression in the rat [26]. Except for the NBRE overlapping the TATA box, none of the proximal promoter NBREs are rigorously conserved across species as previously reported by Wang et al. [27], although Kessler et al. [28] did locate two positionally homologous, remote NBREs in human *TH* and rodent *Th* genes: AAATATCA and AAATGCCA at −8884 and −5364 bp, respectively, in the human *TH* gene. Thus, although proximal sites are the focus of the present report, there remains the possibility that NURR1 and/or 1,25D/VDR act on transcription at DNA loci remote from the proximal promoter region, especially in the case of *TH* mRNA modulation.

Sakurada et al. [29] first demonstrated that NURR1 is a transcriptional activator of *TH* in neural progenitor cells derived from adult rat brain and that the orphan nuclear receptor is able to activate transcription of the *TH* gene by binding to an NBRE located at −873 bp in the promoter-proximal region necessary for midbrain-specific expression. Notably, NURR1 functioned as a monomer, independently of its non-obligatory partner, RXR [29]. Furthermore, Kim et al. [25] probed the most proximal NURR1 site in the rat *TH* gene, at −34 bp, which they refer to as NL1, as well as the −350 bp (NL2) and −873 bp (NL3) sites. Kim and colleagues observed that, surprisingly, NL1 (which overlaps the TATA box) was most crucial for mediating transactivation of rat *Th* by NURR1, despite the fact that both DNase I footprinting and electrophoretic mobility shift assays revealed that NL3 (a perfect consensus sequence match) possessed higher binding affinity for NURR1 than NL1 and NL2. They also demonstrated that the TATA box overlap intrinsic to NL1 apparently was not an impediment in their experiments and did not affect their conclusions. In toto, studies of the rat *Th* proximal promoter region did not yield a definitive indication as to which of the NURR1 elements in this region of the *Th* gene may mediate the proposed action of 1,25D/VDR to secondarily induce *Th*.

In the case of human *TH* gene expression regulation by NURR1, Kim et al. [30], using undifferentiated, dopamine (DA)-like SH-SY5Y neuroblastoma cells cultured with DMEM and 10% fetal calf serum, found that in SH-SY5Y cells, NURR1 dose-dependently stimulated transcription of a human *TH* promoter-proximal construct (−3174 bps) by 7- to 11-fold in this cell context. Notably, the same study reported that NURR1 instead represses this reporter in HB1.F3 human neural stem cells (hNSCs) via a SIRT1-dependent mechanism, illustrating a cell-type-dependent dual function in which NURR1 acts as a transcriptional repressor of *TH* in dopaminergic precursor cells but switches to an activator as those precursors mature into DA-like neurons, a developmental functional switch consistent with the well-established role of NURR1 in driving terminal dopaminergic differentiation. Furthermore, they identified three consensus elements for NURR1 binding: NBRE-A, -B, and -C. NBRE-A corresponds to the −2343 bp element shown in Figure 1A, whereas NBRE-B and NBRE-C represent the −1470 and −855 bp elements in the human *TH* promoter-proximal region, respectively. They employed gel retardation and luciferase assays utilizing human *TH* constructs to demonstrate that NURR1 preferentially bound to NBRE-A (−2343 bp), through which the orphan nuclear receptor mediated *TH* transcriptional activity.

While Kim et al.’s findings establish NBRE-A (−2343 bp) as the functionally dominant NURR1-binding site identified to date in the human *TH* promoter, the regulatory contributions of the remaining proximal NBREs have not been similarly resolved. Neither the −1470 nor −855 bp elements, nor the −35 bp NURR1 site, can be eliminated from consideration as potential contributors to *TH* transcriptional activation by NURR1. Therefore, as with the rat *Th* gene, we are unable at present to pinpoint which NURR1 site in the proximal promoter region of the human *TH* gene is the top candidate to mediate the postulated secondary action of 1,25D/VDR on *TH* expression, and we cannot exclude the possibility that all NURR1 elements in both species cooperate to execute this secondary regulation of *TH/Th* expression.

**Figure 2:**
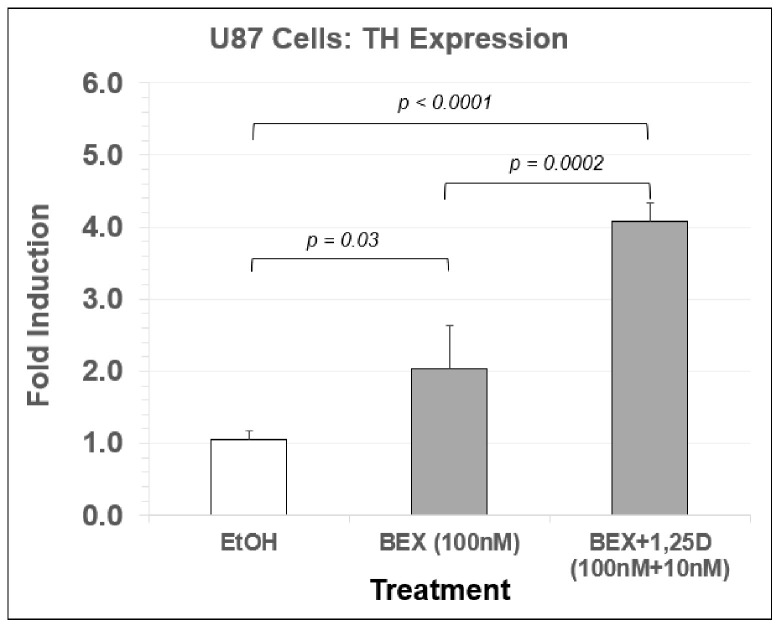
Effects of 1,25D and bexarotene on *TH* expression in human U87 glioblastoma cells. Quantitative PCR analysis of tyrosine hydroxylase (*TH*) mRNA levels following 24 h treatment with ethanol (EtOH; vehicle control), bexarotene (BEX; 100 nM), or a combination of BEX (100 nM) and 1,25-dihydroxyvitamin D (1,25D; 10 nM). *TH* expression is presented as fold induction relative to the EtOH control. Data are shown as mean ± SD from multiple biological replicates. BEX significantly increased *TH* expression compared to control (*p* = 0.03), while co-treatment with BEX + 1,25D further enhanced *TH* expression compared to BEX alone (*p* = 0.0002) and control (*p* < 0.0001), indicating a potentiated effect of combined RXR and VDR signaling.

**Figure 3:**
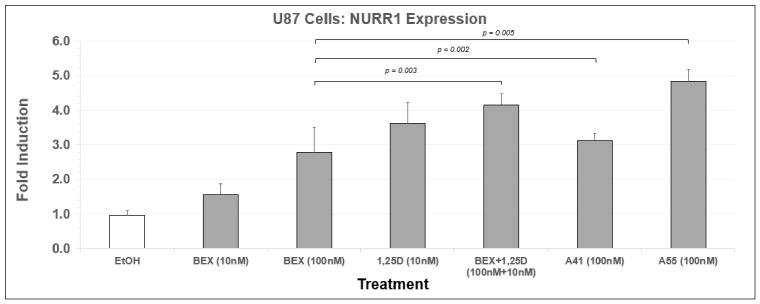
Effects of 1,25D, bexarotene and additional rexinoids on *NURR1* expression in human U87 glioblastoma cells. Quantitative PCR analysis of *NURR1* mRNA levels following 24 h treatment with ethanol (EtOH; vehicle control), bexarotene (BEX; 10 nM and 100 nM), 1,25-dihydroxyvitamin D (1,25D; 10 nM), BEX (100 nM) combined with 1,25D (10 nM), and additional rexinoids (**A41** and **A55**; 100 nM). *NURR1* expression is presented as fold induction relative to the EtOH control. Data are shown as mean ± SD from multiple biological replicates. All treatments significantly increased *NURR1* expression compared to EtOH control, including BEX (10 nM, *p* = 0.0075; 100 nM, *p* = 0.0038), 1,25D (*p* = 0.0007), BEX + 1,25D (*p* < 0.0001), **A41** (*p* = 0.0026), and **A55** (*p* = 0.0008). In addition, as shown above the bars, comparisons between treatments revealed that BEX + 1,25D significantly increased *NURR1* expression compared to BEX alone (*p* = 0.003), while **A41** and **A55** also differed significantly from BEX (100 nM) (*p* = 0.002 and *p* = 0.005, respectively), indicating enhanced RXR-mediated induction of *NURR1*.

**Figure 4:**
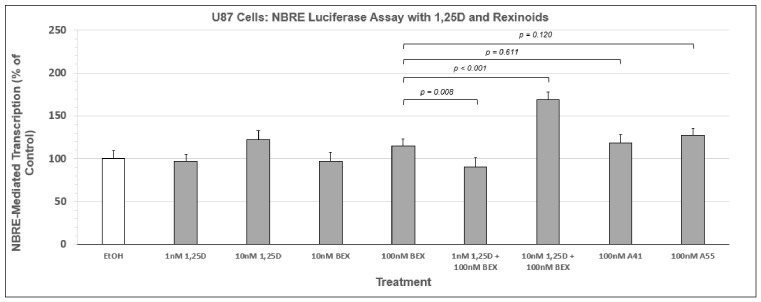
Biological evaluation of 1,25D, bexarotene, and additional rexinoids via an NBRE luciferase-based system in human U87 glioblastoma cells. Cells were transfected with an NBRE-driven luciferase reporter and treated for 18–24 h with ethanol (EtOH; vehicle control), 1,25-dihydroxyvitamin D (1,25D; 1 nM and 10 nM), bexarotene (BEX; 10 nM and 100 nM), combinations of 1,25D and BEX, or additional rexinoids (**A41** and **A55**; 100 nM). Luciferase activity is expressed as percent transcription relative to the EtOH control (set to 100%) and normalized to Renilla activity. Data are presented as mean ± SD from multiple biological replicates. Significant induction (versus EtOH) of NBRE-mediated transcription was observed with 10 nM 1,25D (*p* = 0.014), 100 nM BEX (*p* = 0.005), the combination of 10 nM 1,25D + 100 nM BEX (*p* < 0.0001), as well as rexinoids **A41** (*p* = 0.028) and **A55** (*p* = 0.009), compared to EtOH control. Lower-dose treatments and certain combinations did not reach statistical significance. These results support activation of *NURR1*-dependent transcriptional activity via RXR ligands and enhanced signaling with combined VDR/RXR activation. Additional statistical comparisons and corresponding *p*-values (versus 100 nM BEX) are also shown in the figure, above the bars.

Given the uncertainty surrounding which specific NBRE mediates the NURR1 response, and because NURR1 can engage RXR in a context-dependent manner, we turned to pharmacological tools that broadly engage this NBRE/NURR1/RXR machinery. Selective RXR ligands (rexinoids) provide a tractable experimental entry point for asking whether the NBRE-mediated transcriptional apparatus is functionally engaged at *TH*-relevant regulatory elements, independent of evaluating the central VDR-driven arm of the proposed axis. Since RXR may influence this system both through partnership with NURR1 and through NURR1-independent transcriptional pathways, rexinoid signaling could plausibly potentiate *TH* induction through multiple mechanisms. Because bexarotene is a well-established selective RXR agonist/rexinoid, its induction of *TH* mRNA and enhancement of *TH* mRNA when combined with 1,25D are most parsimoniously interpreted as RXR-linked transcriptional effects, although the present experiments do not prove that every rexinoid-mediated response is exclusively RXR-dependent. We therefore examined the effects of rexinoids on *TH* and *NURR1* expression in human U87 cells. As shown in Figure 2, the prototypical rexinoid, bexarotene (BEX), significantly enhanced *TH* mRNA either alone (2.0-fold) or in combination with 1,25D (4.1-fold), consistent with cooperative action between RXR- and VDR-mediated signaling at the *TH* locus. Furthermore, BEX itself, together with our novel RXR-binding BEX analogs **A41** and **A55**, induced endogenous *NURR1* mRNA in U87 cells (Figure 3) by 2.8-, 3.1-, and 4.8-fold, respectively. Notably, the novel analog **A55** outperformed its parent compound bexarotene at matched 100 nM concentration, identifying **A55** as a more potent NURR1-inducing rexinoid than the bexarotene scaffold from which it was derived. Consistent with this rexinoid-driven elevation of cellular NURR1, both **A41** and **A55** also activated a canonical NBRE-driven luciferase reporter (118% and 127% of the vehicle control, respectively; Figure 4). Importantly, the NBRE motif in this reporter is the same canonical-type element identified in the human *TH* promoter/enhancer region (Figure 1A), so the reporter activation observed in Figure 4 plausibly reflects rexinoid-induced expansion of endogenous NURR1, which then occupies and transactivates NBRE-containing regulatory elements.

This interpretation is further supported by two additional observations. First, titrated *NURR1* overexpression produces dose-dependent activation of the NBRE-driven reporter (Supplementary Figure S2), increasing NBRE-directed reporter activity from near-background levels in the pLUC-empty and pCMV6-empty controls to 21%, 73%, and 100% activity following transfection with 2, 5, and 10 ng pCMV6-Nurr, respectively. This *NURR1* expression-escalation experiment phenocopies the broader directionality of rexinoid-mediated activation of the NBRE-driven reporter (Figure 4) and demonstrates that the NURR1/NBRE reporter platform used in the present study is transcriptionally responsive to increasing *NURR1* expression. Although these data neither validate each individual endogenous *TH* promoter NBRE, nor do they replace ChIP-qPCR or promoter-mutagenesis studies, they provide functional support that the NBRE-based transcriptional system used here is biologically responsive and transcriptionally competent. Together, these findings are consistent with a model in which RXR ligands potentiate *TH* transcription predominantly by expanding the functional pool of NURR1 available to engage canonical NBRE-like elements within the human *TH* promoter/enhancer region, rather than by requiring direct RXR–NURR1 heterodimer binding to a distinct DR-type response element. Accordingly, we use the term cooperative or synergistic in this manuscript to describe enhanced transcriptional output observed when VDR- and RXR-ligand pathways are engaged together, while recognizing that the precise molecular basis of this enhanced response remains to be fully resolved.

**Figure 5:**
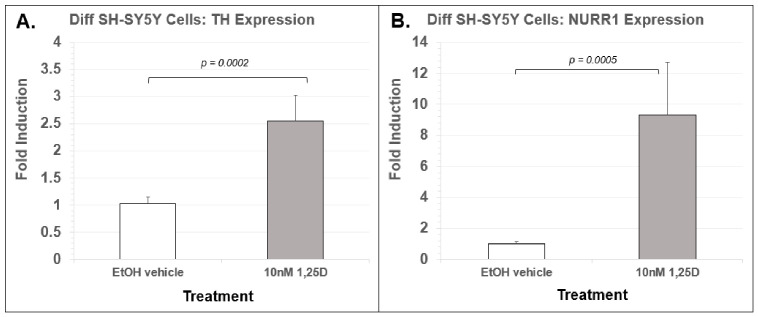
1,25D/VDR induces the mRNA encoding (**A**) TH and (**B**) NURR1 in human differentiated SH-SY5Y neuroblastoma cells. Quantitative PCR analysis of (**A**) tyrosine hydroxylase (*TH*) and (**B**) *NURR1* mRNA levels following 24 h treatment with ethanol (EtOH; vehicle control) or 1,25-dihydroxyvitamin D (1,25D; 10 nM). Gene expression is presented as fold induction relative to the EtOH control. Data are shown as mean ± SD from multiple biological replicates. Treatment with 1,25D significantly increased *TH* expression (*p* = 0.0002) and robustly induced *NURR1* expression (*p* = 0.0005), supporting activation of VDR-mediated transcriptional pathways in differentiated neuronal cells.

**Figure 6:**
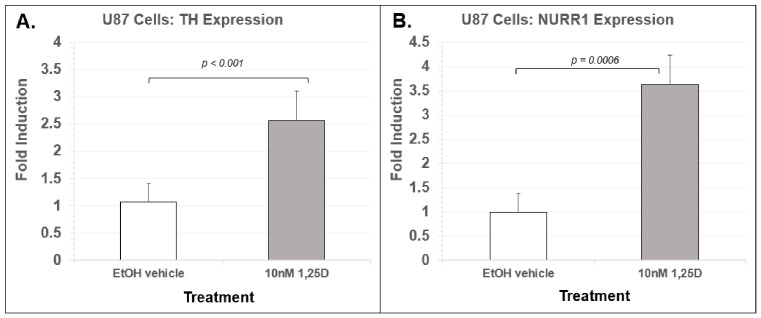
1,25D/VDR induces the mRNA encoding (**A**) TH and (**B**) NURR1 in human U87 glioblastoma cells. Quantitative PCR analysis of (**A**) tyrosine hydroxylase (*TH*) and (**B**) *NURR1* mRNA levels following 24 h treatment with ethanol (EtOH; vehicle control) or 1,25-dihydroxyvitamin D (1,25D; 10 nM). Gene expression is presented as fold induction relative to the EtOH control. Data are shown as mean ± SD from multiple biological replicates. Treatment with 1,25D significantly increased *TH* expression (*p* < 0.001) and *NURR1* expression (*p* = 0.0006), supporting activation of VDR-mediated transcriptional pathways in glioblastoma cells.

Having delineated the contribution of RXR-driven rexinoid signaling to *NURR1* induction and downstream *TH* transcriptional regulation, we next assessed the central VDR-mediated arm of the proposed signaling axis. As illustrated in Figure 5, *TH* mRNA is statistically significantly induced (2.5-fold) and *NURR1* mRNA is induced 9.3-fold when differentiated human SH-SY5Y neuroblastoma cells are treated with 10 nM 1,25D for 24 h. A similar pattern of *TH* (2.6-fold) and *NURR1* (3.6-fold) induction by 1,25D occurs in U87 human glioblastoma cells (Figure 6), although *NURR1* induction by 1,25D is not as prominent in glioblastoma as it is in neuroblastoma cells (compare Figure 5 to Figure 6). The responsiveness of U87 glioblastoma/astrocytoma cells to 1,25D takes on additional translational significance in light of recent neuropathological work by Mazzetti and colleagues, who identified a distinct subpopulation of astrocytes expressing the vitamin D-activating enzyme CYP27B1 exclusively in PD brains; these CYP27B1-positive astrocytes sequestered α-synuclein oligomers and preferentially contacted Lewy-body-negative neurons, suggesting that local astroglial production of bioactive 1,25D contributes to dopaminergic neuroprotection and supporting the relevance of astrocyte-lineage cells as a model for the 1,25D/VDR–NURR1–TH axis we describe here [31]. Nevertheless, the cellular models used here should be interpreted with appropriate limitations. Differentiated SH-SY5Y cells provide a useful human neuronal-like model with dopaminergic/catecholaminergic features, but they are not equivalent to primary midbrain dopaminergic neurons or iPSC-derived dopaminergic neurons. Similarly, U87 cells are not dopaminergic neurons and should not be interpreted as a direct model of dopaminergic neurodegeneration. Rather, the U87 experiments are best viewed as a complementary human neural/glial-lineage transcriptional model for testing VDR/RXR/NURR1-linked regulatory responses. Thus, the strongest dopaminergic interpretation derives from the differentiated SH-SY5Y experiments, whereas the U87 data support the broader concept that 1,25D/VDR- and rexinoid/RXR-linked transcriptional regulation of *NURR1* and *TH* can also occur in a glial-lineage neural cell context.

**Figure 7:**
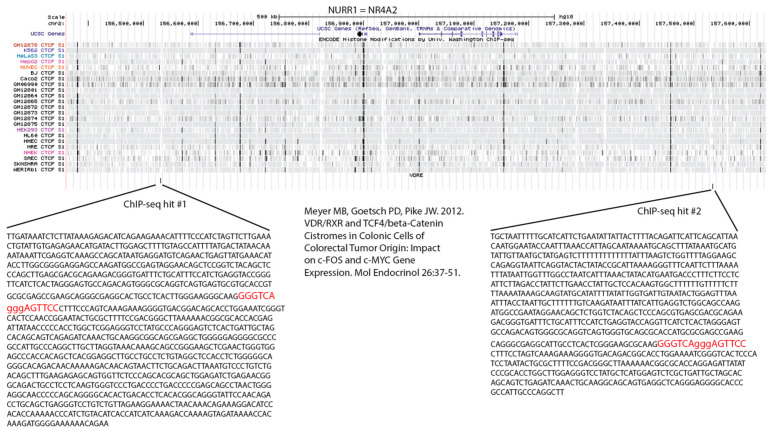
Candidate VDR ChIP-seq-associated VDREs near the human *NURR1* locus. Genomic overview of the human *NURR1* (NR4A2) locus on chromosome 2 (hg18 assembly) displaying VDR-associated ChIP-seq binding data derived from Meyer et al. [33]. The upper panel shows the approximately 1.1 Mb region encompassing the *NURR1* structural gene with UCSC gene annotations and ENCODE histone modification/CTCF binding tracks across multiple cell lines (https://genome.ucsc.edu). Vertical dashed lines indicate two ChIP-seq hits located approximately 200 kb upstream and 300 kb downstream of the *NURR1* gene. Lower panels display expanded nucleotide sequences surrounding each hit, with putative VDREs (GGGTCAgggAGTTCC) highlighted in red. These sequences and their significance to the proposed 1,25D/VDR → *NURR1* signaling model are discussed in the text.

If NURR1 is a secondary mediator of 1,25D/VDR signaling, then VDR-RXR should bind to a VDRE either in the *NURR1* gene or in a driver gene upstream of *NURR1* in terms of signaling. In fact, a ChIP-seq map of the human genome [32] yields a putative VDRE, TGAACCtaaTCCCAT (on the negative strand), located at −3229 bp in the human *NURR1* gene. This proximal VDRE represents a potential site for direct VDR/RXR-mediated transcriptional activation of *NURR1* in response to 1,25D ligand binding. Finally, remote from the human *NURR1* structural gene, two other ChIP-seq hits lie 200 kb upstream and 300 kb downstream [33]. As depicted in Figure 7, the latter two remote VDREs comprise identical sequences: GGGTCAgggAGTTCC. This sequence is a near perfect match to the ideal VDRE, which consists of two hexameric half-elements (GGGTCA and AGTTCC) separated by a three-nucleotide spacer, arranged as a direct repeat with 3 bp spacing (DR3), with the sole exception being a C nucleotide in the n6 position of the 3′ half-element. The presence of identical VDRE sequences at two independent genomic loci flanking *NURR1* suggests that these elements may function cooperatively as distal enhancers, potentially engaging the *NURR1* promoter through chromatin looping mechanisms. Therefore, the potential existence of functional VDREs within or flanking the *NURR1* gene is consistent with primary control of *NURR1* mRNA expression by 1,25D/VDR-RXR, although it does not yet prove this relationship. This bioinformatic evidence aligns with experimental observations of rapid *NURR1* induction by 1,25D in UMR-106 rat osteoblasts reported by Meir et al. [34] and Haussler et al. [35]. Collectively, the identification of three candidate VDREs associated with the human *NURR1* locus (one proximal and two remote) provides a plausible genomic framework through which 1,25D/VDR could directly drive *NURR1* transcription as a primary response gene. This framework is consistent with the robust 9.3-fold induction of *NURR1* mRNA observed in SH-SY5Y cells (Figure 5).

Moreover, Cui et al. [36] originally reported that maternal vitamin D deficiency represses the expression of genes critical for dopamine specification in the embryonic rat mesencephalon, particularly *Nurr1* and *p57Kip2* but not *Pitx3*. Finally, as reported by Latimer et al. [37], high dietary levels of vitamin D induce *Nurr1* mRNA (detected by microarray) in the hippocampus of aged rats, in vivo, while at the same time improving synaptic function and cognitive behavior. This in vivo observation parallels and supports the 1,25D-mediated induction of *NURR1* that we observed in differentiated SH-SY5Y cells (Figure 5) and points to the broader pertinence of 1,25D/VDR signaling for synaptic plasticity and cognitive function. Taken together, the bioinformatic evidence for VDREs at the *NURR1* locus (Figure 7), combined with the functional data demonstrating *NURR1* induction by 1,25D in multiple cell types (Figure 5 and Figure 6) and the in vivo observations from vitamin D-deficient and vitamin D-supplemented animal models, collectively support the hypothesis that *NURR1* may function as a primary transcriptional target of 1,25D/VDR signaling.

The current findings on induction of *TH* mRNA by 1,25D in cultured human cells (Figure 5 and Figure 6) are reinforced by the in vivo research reported by Jiang et al. [2], who demonstrated that chronic administration of 1,25D to rats enhanced *Th* mRNA concentrations in the prefrontal cortex and hippocampus. The data presented in Figure 5 not only confirm the results of Eyles’ group that 1,25D induces *TH* in SH-SY5Y cells [10], but they reveal for the first time that *TH* induction is accompanied by a dramatic (9.3-fold) induction of *NURR1*.

**Figure 8:**
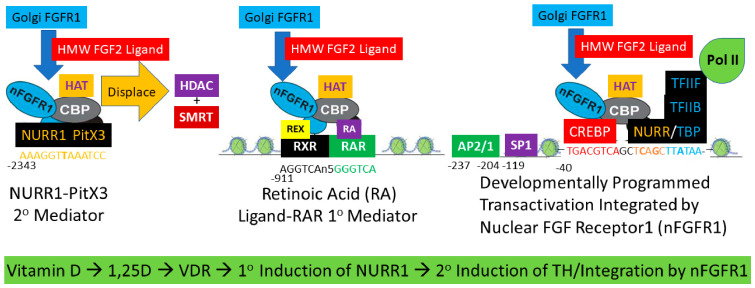
Proposed model for human *TH* gene regulation by 1,25D and retinoids as mediated by various transcription factors. The model depicts three integrated mechanisms of *TH* promoter transactivation, each coordinated by nuclear FGFR1 (nFGFR1) and its co-integrator CBP. (**Left panel**): *NURR1*, cooperating with Pitx3, binds to the NBRE at −2343 bp in the human *TH* promoter, displacing the HDAC/SMRT co-repressor complex and recruiting histone acetyltransferase (HAT) activity to activate transcription. This represents the proposed secondary response to 1,25D, in which VDR-induced *NURR1* expression is hypothesized to increase NURR1-linked transcriptional activity at the *TH* promoter. (**Middle panel**): Retinoic acid (RA) or rexinoid (REX) acting through RAR/RXR heterodimers binds to the DR5-type RARE at −911 bp, with additional contributions from AP-2, AP-1, and SP1 elements in the intervening promoter region. This represents a parallel, ligand-dependent primary mechanism for *TH* induction by retinoids. (**Right panel**): Developmentally programmed transactivation at the core promoter, where CREB, NURR1, and TBP converge at the conserved CRE/AP-1 site (−40 bp) and adjacent TATA box (−30 bp), recruiting RNA Polymerase II and general transcription factors (TFIIB, TFIIF) to initiate *TH* mRNA synthesis. In all three scenarios, nFGFR1 is released from the Golgi in response to high-molecular-weight FGF2 ligand and translocates to the nucleus, where it serves as an integrative platform coordinating CBP-mediated chromatin remodeling with promoter-bound transcription factors. The bottom summary depicts the overall signaling pathway: vitamin D → 1,25D → VDR → primary induction of NURR1 → secondary induction of TH, with integration by nFGFR1.

To further support the functional connection between *NURR1* expression and endogenous *TH* transcription, we have included *NURR1* expression plasmid titration data as Supplementary Figure S1. In U87 cells, escalating amounts of pCMV6-Nurr increased endogenous *TH* mRNA in a dose-dependent manner, from 1.0-fold in the pCMV6-empty vector control to 1.7-, 3.8-, and 5.1-fold following transfection with 2, 5, and 10 ng pCMV6-Nurr, respectively. Although this expression-escalation strategy does not establish that NURR1 is required for the full 1,25D-mediated *TH* response, it addresses the same regulatory relationship from the complementary gain-of-function direction and supports the conclusion that experimentally increasing NURR1 is sufficient to enhance endogenous *TH* mRNA. Together with prior evidence that NURR1 transfection dose-dependently stimulates hTH-3174 promoter activity by 7- to 11-fold [30], we hypothesize that NURR1 is a mediator of the secondary action of 1,25D/VDR to enhance *TH* transcription and present the model pictured in Figure 8. The model integrates three distinct but coordinated regulatory mechanisms operating at different regions of the human *TH* promoter, each unified by the integrative signaling platform provided by nuclear FGFR1 (nFGFR1) and its co-activator CBP. Supporting this hypothetical model is the observation by Luan et al. [38] that developmental vitamin D deficiency attenuates *Nurr1* and *Th* expression in postmitotic dopamine neurons in rat mesencephalon.

A molecular foundation for this hypothesis is provided by the existence of four *NURR1* element binding sites in the proximal promoter DNA sequence of the human *TH* gene: one overlapping the TATA box at −30 bp and three more at −855, −1470 and −2343 bp. We postulate that NURR1 docks on one or more of these sites, most likely the −30 and −2343 bp elements, to mediate the secondary response of *TH* gene expression to 1,25D/VDR-RXR by attracting CBP and nFGFR1 to signal an enhancement of *TH* mRNA synthesis as depicted in Figure 8. In this context, NURR1 binding at the −2343 bp NBRE is proposed to displace pre-existing HDAC/SMRT co-repressor complexes, thereby permitting recruitment of histone acetyltransferase (HAT) activity and a local transition from a repressive to an active chromatin state. Concurrently, Pitx3 may cooperate with NURR1 at this locus, consistent with the juxtaposition of Pitx3 response elements and NBREs observed in the human *TH* promoter (Figure 1A) and with the established cooperative function of these two factors in determining dopaminergic neuron cell fate [16,25].

In addition to this NURR1-mediated secondary pathway, the model (Figure 8) incorporates a parallel mechanism through which retinoic acid, acting via RAR/RXR heterodimers at the DR5-type RARE located at −911 bp, can independently drive *TH* transcription. This is consistent with the known stimulation of *TH* gene expression by retinoic acid [18] and with the ability of RXR to participate in multiple heterodimeric partnerships (including VDR/RXR, RAR/RXR, and NURR1/RXR) that converge on the *TH* promoter. The rexinoid data presented herein (Figure 2, Figure 3 and Figure 4) support the concept that RXR serves as a shared co-regulatory partner capable of potentiating *TH* expression through both NURR1-dependent and retinoid-dependent pathways.

At the core promoter, the model depicts the convergence of CREB, NURR1, and TBP at the conserved CRE and TATA box region, where they cooperate to recruit RNA Polymerase II and general transcription factors for transcriptional initiation. This arrangement is consistent with the known role of the CRE/AP-1 site at −40 bp as a docking point for nFGFR1, which cooperates with NURR1 in the postmitotic development of mesencephalic dopaminergic neurons [24].

This model for *TH* control by 1,25D is based on the thesis of Stachowiak [18] and specifically on the work of Baron et al. [24], who studied cooperation of nFGFR1 and NURR1 experimentally to develop a novel interactive mechanism for postmitotic development of mesencephalic dopaminergic neurons. Importantly, nFGFR1 is proposed to function as a master integrative platform in this system: upon release from the Golgi in response to high-molecular-weight FGF2 ligand, nFGFR1 translocates to the nucleus where it coordinates CBP-mediated chromatin remodeling with the activities of promoter-bound transcription factors across multiple regulatory regions of the *TH* gene. Thus, the model posits that 1,25D/VDR signaling feeds into a pre-existing developmental program for *TH* regulation by expanding the pool of NURR1 available to engage this nFGFR1-coordinated transcriptional machinery.

Although TH has been studied extensively as a pivotal enzyme in the biosynthetic pathway leading to dopamine, norepinephrine, and epinephrine, including detailed analyses of its proximal promoter region [27,28], the transcriptional mechanism by which 1,25D/VDR signaling regulates TH has remained incompletely defined. Induction of *TH* expression by 1,25D was first reported in bovine adrenal medullary cells by Puchacz and colleagues [19] and was subsequently extended to dopaminergic-lineage cells by Cui et al. [10], who demonstrated dose- and time-dependent TH induction in VDR-transfected SH-SY5Y cells, corroborated this response in a developmental vitamin D-deficient model in vivo, and proposed N-cadherin as a candidate mediator. The experiments reported here reproduce 1,25D-mediated TH induction in differentiated SH-SY5Y and U87 cells (Figure 5 and Figure 6) but, more importantly, identify a distinct regulatory layer: a NURR1-linked transcriptional program operating at the level of *TH* gene expression, rather than solely through cell adhesion or broader differentiation cues.

This dopaminergic differentiation framework was developed further by Pertile and colleagues, who, using a VDR-overexpressing SH-SY5Y subline (SH-SY5Y/VDR+) together with primary mesencephalic cultures and mesencephalic/striatal explant co-cultures, reported that 1,25D promotes a coordinated set of dopaminergic features, including neurite outgrowth, neurite branching, presynaptic protein redistribution, and functional dopamine release [39]. Collectively, the prior work by Puchacz et al., Cui et al., Jiang et al. and Pertile et al. indicates that 1,25D/VDR-linked effects in catecholaminergic/dopaminergic systems can extend beyond mRNA-level changes to include TH-associated cellular responses, dopaminergic differentiation, altered dopamine-related metabolites, and functional dopamine release. The present study does not itself measure TH or NURR1 protein levels or catecholamine output; instead, it defines a transcriptional *NURR1*-centered regulatory framework that can be tested in future protein-level and functional dopamine/catecholamine studies. Although these prior studies establish 1,25D as a modulator of catecholaminergic/dopaminergic phenotype, the transcriptional architecture driving TH induction itself was not resolved. The present study addresses this gap directly by identifying NURR1 as a proximal transcriptional effector through which 1,25D/VDR signaling converges on *TH*, providing a mechanism that is complementary to (but mechanistically distinct from) the N-cadherin/differentiation framework and one that plausibly underlies the phenotypic responses reported in those earlier studies.

The N-cadherin framework proposed by Cui et al. [10] rests on two key observations. First, developmental vitamin D deficiency phenocopies regional N-cadherin ablation in the mesencephalon, and second, 1,25D induces N-cadherin expression [10,40]. The mechanistic model developed herein (Figure 8) departs from that framework in proposing NURR1 as the primary 1,25D-induced transcriptional effector, with increased *NURR1* expression hypothesized to enhance NURR1-linked transcriptional activity at *TH* regulatory regions. While the N-cadherin hypothesis invokes a cell-adhesion signaling intermediate, our model positions NURR1, itself a nuclear receptor and an established transcriptional activator of *TH*, as a more direct link between VDR activation and *TH* gene induction. We cannot, however, exclude a more complex signaling architecture in which N-cadherin and NURR1 both contribute, potentially alongside additional candidate transcription factors such as MZF1, which has notably been reported to upregulate N-cadherin mRNA in developing osteoblasts [41].

Furthermore, in a striking convergence of concepts, 1,25D has been shown recently to induce N-cadherin in HN9.10e embryonic hippocampal cells and in hippocampus of a MPTP-induced Parkinson’s disease mouse model [40]. Indeed, 1,25D/VDR appears capable of inducing both NURR1 and N-cadherin, two candidate intermediaries through which 1,25D may indirectly upregulate *TH* expression in the CNS, thereby identifying transcriptional and differentiation-linked mechanisms with potential relevance to dopaminergic biology and Parkinson’s disease-related pathways [42]. It is conceivable that these two pathways are not mutually exclusive but rather operate in parallel or converge downstream, with NURR1 providing direct transcriptional activation at the *TH* promoter while N-cadherin contributes through cell-adhesion-mediated signaling that may influence dopaminergic neuron survival and connectivity.

Moreover, recent studies demonstrate that VDR signaling exerts neuroprotective effects in dopaminergic systems by reducing oxidative stress, preserving mitochondrial function, and limiting microglial-mediated inflammation, providing additional support for the role of vitamin D signaling in PD pathophysiology [43]. Adding direct in vivo causal support for this anti-inflammatory dimension of 1,25D action, Xie and colleagues demonstrated in a 6-OHDA-induced hemiparkinsonian mouse model that calcitriol administration restrains microglial M1 polarization, expands splenic regulatory T-cell (Treg) populations, and preserves dopaminergic neurons, with antibody-mediated Treg depletion abolishing the protective effect, thereby establishing coordinated central microglial repolarization and peripheral Treg-mediated immunomodulation as causal mediators of 1,25D-driven neuroprotection in a recognized PD model [44].

Consistent with these mechanistic findings, clinical analyses reported by Xia and Zhou [45] demonstrate reduced circulating vitamin D levels in patients with PD, further supporting a link between vitamin D status and dopaminergic dysfunction. Going beyond observational correlation to controlled interventional evidence, a recent randomized, double-blind, placebo-controlled trial by Li and colleagues reported that three months of vitamin D_3_ supplementation in vitamin D-deficient PD patients significantly raised serum 25(OH)D_3_ levels, reduced peripheral Th17 cells, expanded regulatory T-cell populations, and produced measurable improvements in motor function (UPDRS and UPDRS-III, *p* < 0.001)—outcomes not observed in the placebo arm—providing human-trial evidence that vitamin D-mediated rebalancing of the Th17/Treg axis can translate into clinical benefit in PD, directly paralleling the Treg-dependent neuroprotective mechanism established preclinically [46]. A contemporaneous meta-analysis by Xu and colleagues pooling eight randomized controlled trials (646 PD patients) provides the broader pooled-evidence counterpoint. Namely, across this pooled trial base, vitamin D supplementation did not significantly improve UPDRS-III, timed-up-and-go, or short-distance walk test outcomes. The one positive signal was a statistically significant ~25 m improvement in 6 min walking distance. Taken together, these results indicate that motor benefits in unselected PD populations are likely modest and may depend on baseline vitamin D status, dosing, treatment duration, and clinical context. Consistent with the Li et al. findings, the strongest effects may be most detectable in deficiency-enriched cohorts, although this interpretation will require confirmation in larger, prospectively stratified trials [47]. Such variable trial outcomes are also aligned with (and arguably predicted by) a mechanistic framework in which the clinical impact of vitamin D supplementation depends on VDR availability and function (potentially modulated by polymorphisms such as FokI), baseline *NURR1* expression, and the saturation state of the downstream dopaminergic transcriptional pathway. Population heterogeneity in this signaling axis, rather than the absence of effect, may therefore best account for the divergent results observed across supplementation trials. This context-dependent interpretation is consistent with our recent VDR-RXR transcriptional synergy study [48], which showed that combined vitamin D/rexinoid responses are influenced by cellular background, VDRE architecture, VDR expression/polymorphic context, ligand concentration, and rexinoid structure. Thus, these in vivo and clinical studies are best viewed as biological context for the present transcriptional model, rather than as direct validation of the specific 1,25D/VDR–NURR1–TH mechanism proposed here.

Collectively, these findings support a model in which 1,25D/VDR signaling induces NURR1 as a primary transcriptional event, which in turn drives secondary activation of *TH* gene expression through engagement of NBRE-containing regulatory elements. This integrated signaling axis provides a mechanistic framework linking vitamin D biology to dopaminergic gene regulation and highlights a potentially important pathway for maintaining neuronal function and resilience in neurodegenerative disease. Future studies employing NURR1 loss-of-function approaches, chromatin occupancy assays, and in vivo validation in dopaminergic systems will be essential to definitively establish the causal role of NURR1 in mediating the TH-inductive response to 1,25D.

## 4. Conclusions

In summary, our findings demonstrate that 1,25D significantly induces *TH* mRNA expression in human neural cell models, supporting a transcriptional role for vitamin D signaling in the regulation of TH-linked dopaminergic gene programs. We provide evidence supporting a model in which this effect is mediated, at least in part, through primary VDR-driven induction of NURR1, an orphan nuclear receptor and key transcriptional regulator of dopaminergic neuron development and function. This proposal is supported by three convergent lines of evidence: (1) parallel induction of *TH* and *NURR1* by 1,25D in both differentiated SH-SY5Y neuroblastoma and U87 glioblastoma cells; (2) comparative identification of candidate NBREs across human, mouse, and rat *Th* proximal promoters, including a positionally conserved NBRE overlapping the TATA box; and (3) identification of candidate VDREs at the human *NURR1* locus through interrogation of published VDR ChIP-seq/cistrome datasets.

The conceptual advance of the present study is not the isolated observation that vitamin D can influence dopaminergic biology, nor the isolated observation that NURR1 can regulate *TH* transcription, as both concepts are supported by the prior literature. Rather, the advance is the integration of these previously separate observations into a unified transcriptional framework in which 1,25D/VDR signaling induces NURR1, NURR1 functions as a plausible secondary mediator of TH induction, and RXR/rexinoid signaling modulates this NURR1-linked transcriptional axis. In this way, the study connects vitamin D/VDR biology, NURR1-dependent TH regulation, and rexinoid/RXR pharmacology into a single experimentally supported model that generates specific testable predictions for future mechanistic studies.

The observed upregulation of both *TH* and *NURR1* is reinforced by the ability of rexinoids, including bexarotene and the novel analog **A55** (which outperforms its parent compound at matched concentration) to further enhance *TH* transcription via RXR-dependent pathways. Notably, independent in vivo studies comparing **A55** with bexarotene in murine mammary and lung cancer models reported that **A55** produced greater tumor reduction than bexarotene while avoiding the triglyceride elevation observed with the parent drug, with triglyceride levels remaining comparable to vehicle controls at the same dose [49]. Although these findings were obtained outside the dopaminergic context, they further support **A55** as a pharmacologically attractive RXR-directed scaffold and strengthen the rationale for exploring rexinoid modulation of NURR1-linked transcriptional programs. Together, these findings support a model in which a coordinated 1,25D/VDR–NURR1–RXR signaling axis expands the functional pool of NURR1 to drive transcriptional activation at NBRE-containing regions of the *TH* gene.

Collectively, these data also position NURR1 as a central transcriptional intermediary linking vitamin D signaling to dopaminergic gene regulation, while leaving open the possibility that an N-cadherin–mediated pathway, also induced by 1,25D, operates in parallel to support dopaminergic neuron survival and connectivity. Importantly, this framework may also help contextualize the heterogeneous outcomes observed in vitamin D supplementation trials in PD, in which clinical benefit appears greatest in deficiency-enriched cohorts and is plausibly modulated by VDR availability, VDR polymorphisms, and baseline *NURR1* expression. Given the established roles of dopamine in motor control, cognition, and neurodegenerative disease, these findings provide mechanistic insight into how vitamin D may modulate dopaminergic transcriptional programs, while identifying the 1,25D/VDR–NURR1–RXR axis as a candidate transcriptional pathway for future mechanistic and translational studies in Parkinson’s disease-relevant systems.

Some limitations of the present study should be emphasized. First, although the data support a model in which 1,25D/VDR induction of NURR1 contributes to secondary induction of TH, NURR1 loss-of-function experiments will be required to determine whether NURR1 is necessary for the full TH response to 1,25D. Second, the candidate VDREs associated with the *NURR1* locus and the candidate NBREs within *TH* regulatory regions require further validation by chromatin occupancy, promoter-reporter, and/or mutagenesis-based approaches. Third, the present study focuses primarily on transcriptional regulation and does not directly measure TH or NURR1 protein levels, dopamine production, catecholamine biosynthesis, neuronal survival, or neuroprotection in a Parkinson’s disease model. Finally, although differentiated SH-SY5Y and U87 cells provide useful human neural cell contexts for evaluating VDR/RXR/NURR1-linked transcriptional regulation, future studies in iPSC-derived dopaminergic neurons, primary mesencephalic cultures, and/or in vivo Parkinson’s disease models will be needed to determine how broadly this proposed axis operates in more physiologically relevant dopaminergic systems.

Beyond these mechanistic limitations, future work should determine whether targeted modulation of the 1,25D/VDR–NURR1–RXR axis can influence dopaminergic resilience or therapeutic responsiveness in neurodegenerative contexts involving NURR1 dysfunction or dopaminergic decline. Such studies may also help define whether vitamin D status, VDR signaling capacity, RXR-ligand responsiveness, or baseline NURR1 activity stratify cellular or organismal responses to this regulatory pathway. In this way, the transcriptional framework proposed here provides a foundation for testing how endocrine, nuclear receptor, and dopaminergic gene-regulatory programs intersect in Parkinson’s disease-relevant biology.

## Data Availability

The original contributions presented in this study are included in the article/Supplementary Material. Further inquiries can be directed to the corresponding author.

## Supplementary Materials

The following supporting information can be downloaded at: https://www.mdpi.com/article/10.3390/cells15131210/s1, Figure S1: NURR1 expression plasmid titration increases endogenous TH mRNA in U87 cells; Figure S2: NURR1 expression plasmid titration activates an NBRE-driven luciferase reporter in U87 cells.
