# Supplementary material for: 1,25-Dihydroxyvitamin D Induces a NURR1–Tyrosine Hydroxylase Transcriptional Axis Modulated by Rexinoid/RXR Signaling in Parkinson’s Disease-Relevant Human Neural Cell Models
Source: Cells. 2026 Jul 3;15(13):1210. doi: 10.3390/cells15131210 (PMC13359966; doi:10.3390/cells15131210)
Supplement: Supplementary file 1 [file cells-15-01210-s001.zip › cells-4356003-supplementary.pdf]

# Supplementary Figures

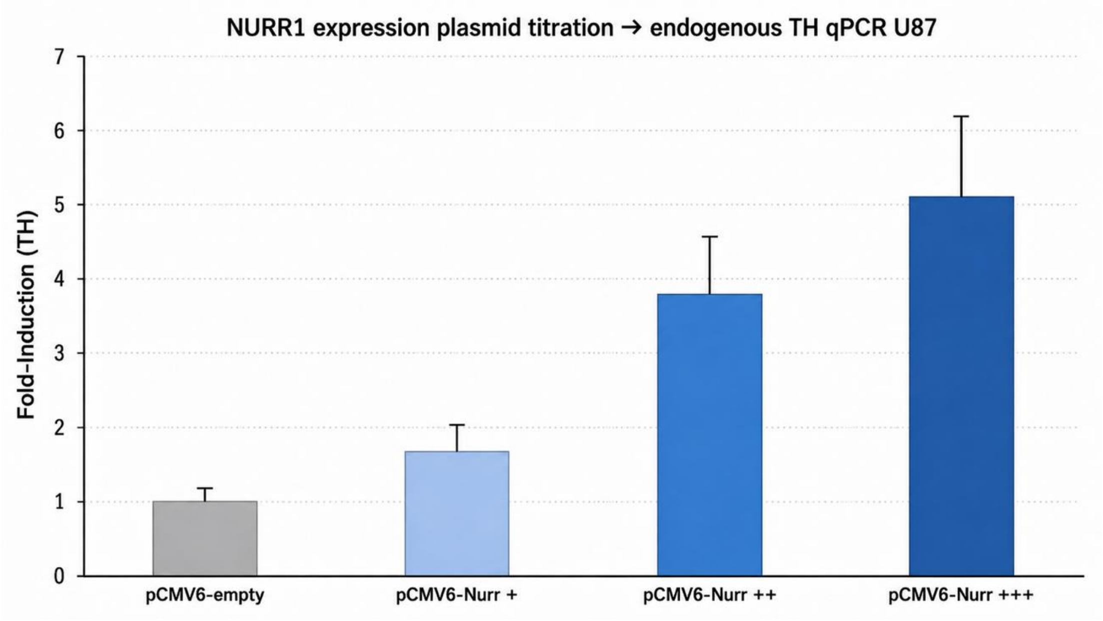


**Figure S1. NURR1 expression plasmid titration increases endogenous TH mRNA in U87 cells.** U87 cells were transfected with the pCMV6-empty parent expression vector or increasing amounts of pCMV6-Nurr (NURR1) expression plasmid. The pCMV6-empty control received 10 ng of empty vector. For the NURR1 titration, pCMV6-Nurr was transfected at 2 ng (+), 5 ng (++), or 10 ng (+++), with total pCMV6-based expression vector held constant at 10 ng in each condition by supplementing with pCMV6-empty as needed. Endogenous TH mRNA expression was quantified by real-time qPCR using the same RNA isolation, cDNA synthesis, normalization, and ΔΔCt-based analysis procedures described in the Methods. TH expression is presented as fold induction relative to the pCMV6-empty control, which was set to 1.0. Increasing NURR1 expression plasmid produced a dose-dependent increase in endogenous TH mRNA. Data are from three independent biological replicates with four technical replicates per treatment-transfection group. Bars represent mean values, and error bars indicate +SD.

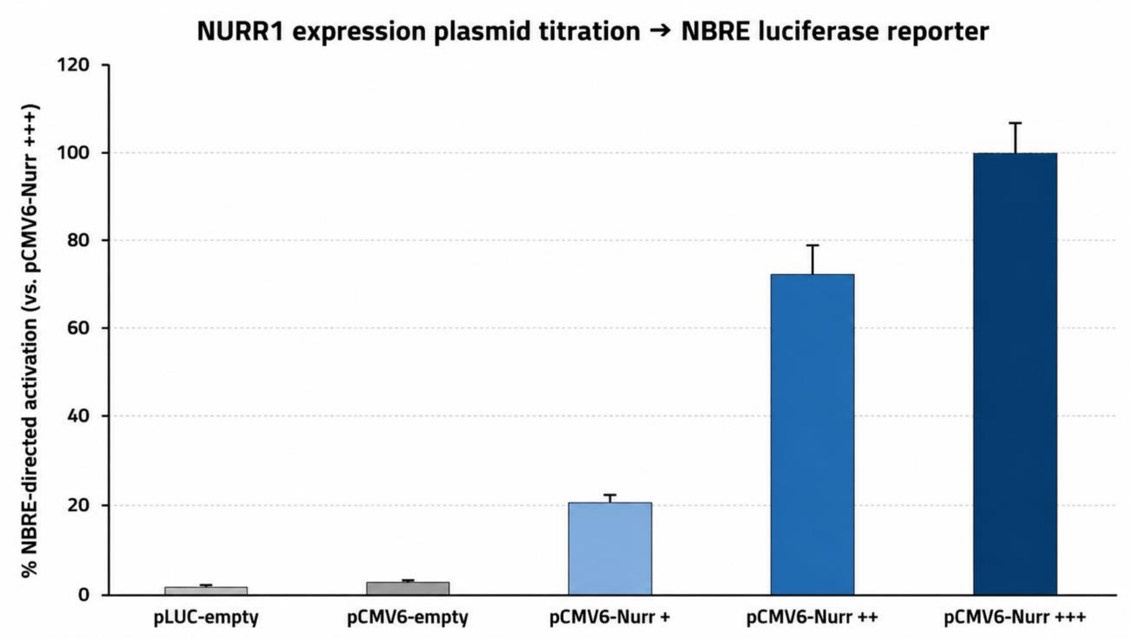


**Figure S2. NURR1 expression plasmid titration activates an NBRE-driven luciferase reporter in U87 cells.** U87 cells were transfected with either the pLUC-empty parent luciferase vector lacking NBRE response elements or an NBRE-driven luciferase reporter (NBRE-LUC), together with pCMV6-empty or increasing amounts of pCMV6-Nurr (NURR1) expression plasmid. The pLUC-empty and NBRE-LUC reporter plasmids were each transfected at 250 ng. The pLUC-empty condition was paired with 10 ng pCMV6-Nurr (+++) as a reporter-backbone control in the presence of maximal NURR1 expression. For NBRE-LUC conditions, the pCMV6-empty control received 10 ng of empty expression vector, while pCMV6-Nurr was transfected at 2 ng (+), 5 ng (++), or 10 ng (+++), with total pCMV6-based expression vector held constant at 10 ng in each condition by supplementing with pCMV6-empty as needed. Luciferase activity was measured using the same Firefly/Renilla normalization procedure described in the Methods. NBRE-directed activation is expressed relative to the NBRE-LUC + pCMV6-Nurr +++ condition, which was set to 100%. Increasing NURR1 expression plasmid produced dose-dependent activation of the NBRE-driven reporter. Data are from three independent biological replicates with four technical replicates per treatment/transfection group. Bars represent mean values, and error bars indicate +SD.
